# Supplementary material for: Direct observation of one-dimensional disordered diffusion channel in a chain-like thermoelectric with ultralow thermal conductivity
Source: Nat Commun. 2021 Nov 18;12:6709. doi: 10.1038/s41467-021-27007-y (PMC8602660; doi:10.1038/s41467-021-27007-y)
Supplement: Supplementary file 1 — Supplementary Information [file 41467_2021_27007_MOESM1_ESM.pdf]

Supplementary Information

for

Direct observation of one-dimensional disordered diffusion channel in a chain-like thermoelectric with ultralow thermal conductivity

Jiawei Zhang[1], Nikolaj Roth[1], Kasper Tolborg[1], Seiya Takahashi[2], Lirong Song[1], Martin Bondesgaard[1], Eiji Nishibori[2], Bo B. Iversen[1*]*

[1]Center for Materials Crystallography, Department of Chemistry and iNANO, Aarhus University, DK-8000 Aarhus, Denmark

[2]Faculty of Pure and Applied Sciences and Tsukuba Research Center for Energy Materials Science (TREMS), University of Tsukuba, Tsukuba 305-8571, Japan

*Correspondence to: jiaweizhang@chem.au.dk, bo@chem.au.dk

Including:

Supplementary Notes 1-2

Supplementary Tables 1-8

Supplementary Figures 1-18

Supplementary References 1-7

Supplementary Note 1. Structure refinements

Previously reported structure models[1,2] generally assume the three atomic sites in InTe to be fully occupied (i.e., the full occupancy model), which simply overlooks the structural disorder. In this study, we conducted structure refinements with different structure models including the full occupancy model, vacancy model, and two interstitial model (see Supplementary Tables 5-7). The two interstitial model not only gives the best fit to the single-crystal synchrotron X-ray diffraction data, but also shows a composition consistent with the experimental observation (see Table 1 in the main text). For structure refinements with the two interstitial model, the interstitial indium sites refine best with the anisotropic atomic displacement parameters (ADPs). As the refined site occupancies for In^{3+} and Te are generally very close to unity (i.e. 1.0008(15) and 0.9995(14) respectively for In^{3+} and Te at 25 K), we thereby only refined the site occupancy of In^{1+} and indium interstitials. After the refinements of the site occupancy and isotropic ADP, the site occupancy was fixed during the anisotropic ADP refinements to avoid any possible correlation between the anisotropic ADP and site occupancy of the indium interstitial sites. According to the MEM density at 25 K (see Fig. 2a in the main text), the electron density peaks at two interstitial sites are comparable. As a result, the structure refinements generally give nearly the same site occupancies and ADPs for the two interstitial sites. To reduce the refinement parameters for simplicity, the ADPs of the two interstitial indium sites were constrained to be the same during the refinements. As the location of the interstitial atoms is typically stable, we kept the positions and site occupancies of indium interstitials from 25 K and moved to the refinements at other temperatures. The two interstitial model refinements are only possible up to 200 K. As the temperature increases above 200 K, the refinements show a clear divergence with unphysically large ADP of interstitial indiums along the *c* direction. This is consistent with the dynamic behavior of interstitial indiums as revealed by the 1D continuous In^{1+} channel of the MEM electron density (see Fig. 3 in the main text). In fact, the electron density connection through the 1D In^{1+} channel already starts between 100 and 200 K (Supplementary Fig. 13), which accounts for a clear increase in U_{33} of the interstitial indiums at 100-200 K (see Fig. 3e in the main text).

Debye temperature of InTe was estimated using the Debye expression[3,4]:

$$U_{eq}(T) = \frac{3h^2T}{4\pi^2 m k_B \Theta_D^2}\left(\frac{T}{\Theta_D}\int_0^{\Theta_D/T}\frac{x}{e^x-1}dx + \frac{\Theta_D}{4T}\right) + d^2 \,. \quad (1)$$

Here h is the Planck constant, T is the absolute temperature, m is the mass of the atom, k_B is the Boltzmann constant, Θ_D is the Debye temperature, and d is a disorder parameter.

Supplementary Note 2. Residual density analysis

The actual optimal stopping criterion χ^2 for MEM density analysis is dependent on the quality of the data and the structure refinement program. This is typically because of the standard deviation determination, which usually differs with different programs. To ensure the optimum χ^2 value for obtaining the optimal MEM density, residual density analysis was performed for all temperatures according to the method proposed by Bindzus and Iversen[5]. Residual densities were calculated by the inverse Fourier transformation of $F_{obs}(\mathbf{H}) - F_{MEM}(\mathbf{H})$ for each reciprocal lattice vector **H**. The optimum χ^2 value was determined with the value where the residual density has the most parabolic fractal dimension distribution[5,6], that is, coefficients of determination R^2, obtained by fitting the fractal dimension distributions to a parabolic function, reaches the optimal value (see Supplementary Table 8 and Supplementary Figs. 9 and 10).

Supplementary Table 1 | Chemical composition of InTe single crystal determined by ICP-OES.

	In (atom%)	Te (atom%)	x in In_xTe
ICP-OES	49.54 (0.11)	50.46 (0.42)	0.98 (0.01)

Supplementary Table 2 | Debye temperatures of InTe estimated by the Debye expression using the temperature-dependent U_{eq} from different structure models.

Model	$\Theta_D(In^{3+})$ (K)	$\Theta_D(In^{1+})$ (K)	$\Theta_D(In_i)$ (K)	Θ_D(Te) (K)	Θ_D(Average) (K)
Full occupancy model	136(2)	85(1)	-	133(2)	114(1)
Vacancy model	136(2)	88(1)	-	133(2)	116(1)
Two interstitial model	146(0)	92(2)	112(4)	143(0)	124(1)

Supplementary Table 3 | Experimental details of the single-crystal synchrotron X-ray diffraction data of InTe collected at 25-700 K.

T (K)	25	100	200	300	400	500	600	700
λ (Å)	0.2480	0.2480	0.2480	0.2480	0.2480	0.2509	0.2509	0.2509
$(\sin\theta/\lambda)_{max}$ (Å^{-1})	1.67	1.83	1.50	1.28	1.15	1.24	1.00	0.97
$N_{measured}$	89683	102115	83374	67601	52896	110481	22237	20489
N_{unique}	2723	3532	2029	1307	971	1255	680	630
N_{unique} ($I>3\sigma$)	2492	3046	1746	1159	848	989	562	504
Redundancy	32.9	28.9	41.1	51.7	54.5	88.0	32.7	32.5
Completeness (%)	99.6	98.7	99.9	100	100	100	100	100
R_{merge} (%)	4.13	3.68	3.83	3.86	3.95	3.91	4.04	4.93
R_σ (%)	2.49	2.45	2.40	2.39	2.40	2.36	2.57	2.99
$\langle I/\sigma \rangle$	30.49	24.89	27.36	29.48	29.38	24.02	25.64	22.28

Supplementary Table 4. Atomic positions and atomic thermal displacement parameters for the two interstitial model of InTe at 25 K.

Atom	Wyckoff position	*x*	*y*	*z*	Occupancy	U_{11} (Å^2)	U_{33} (Å^2)
In^{1+}	4a	0.5	0.5	0.25	0.8948(12)	0.006051(16)	0.02708(6)
In^{3+}	4b	0	0.5	0.25	1	0.004020(11)	0.002103(16)
Te	8h	0.183636(3)	0.316364(3)	0.5	1	0.003422(10)	0.002866(14)
In_i(1)	8f	0.5	0.5	0.5463(4)	0.0156(5)	0.0049(2)	0.0041(5)
In_i(2)	8f	0.5	0.5	0.6369(4)	0.0155(5)	0.0049(2)	0.0041(5)

Supplementary Table 5 | Refinement details with the full occupancy model of InTe based on the single-crystal synchrotron X-ray diffraction data at 25-700 K. The occupancies of the three atomic sites were set to unity.

T (K)	25	100	200	300	400	500	600	700
a (Å)	8.3685(3)	8.3802(3)	8.3996(1)	8.4224(1)	8.4458(2)	8.4697(2)	8.5004(3)	8.5249(3)
c (Å)	7.1212(2)	7.1212(2)	7.1260(2)	7.1339(2)	7.1417(2)	7.1500(2)	7.1562(4)	7.1687(5)
$U_{11}(In^{1+})$ (Å^2)	0.00693(4)	0.01611(4)	0.02947(9)	0.04318(16)	0.0577(3)	0.0730(4)	0.0904(7)	0.1088(10)
$U_{33}(In^{1+})$ (Å^2)	0.0296(1)	0.04657(17)	0.0653(3)	0.0846(5)	0.1078(9)	0.1334(13)	0.1491(18)	0.187(2)
$U_{eq}(In^{1+})$ (Å^2)	0.01447(5)	0.02626(6)	0.04141(12)	0.05701(19)	0.0744(3)	0.0932(5)	0.1100(7)	0.1348(10)
$U_{11}(In^{3+})$ (Å^2)	0.00389(2)	0.00855(2)	0.01540(3)	0.02279(5)	0.03065(8)	0.03920(10)	0.04882(18)	0.0590(3)
$U_{33}(In^{3+})$ (Å^2)	0.00205(3)	0.00416(2)	0.00751(3)	0.01119(5)	0.01495(8)	0.01930(9)	0.02406(16)	0.0286(2)
$U_{eq}(In^{3+})$ (Å^2)	0.00328(2)	0.00709(1)	0.01277(2)	0.01892(3)	0.02542(5)	0.03256(6)	0.04056(10)	0.04887(15)
U_{11}(Te) (Å^2)	0.00331(2)	0.00701(1)	0.01257(3)	0.01857(4)	0.02505(7)	0.03196(8)	0.03986(13)	0.0481(2)
U_{33}(Te) (Å^2)	0.00274(3)	0.00611(2)	0.01109(3)	0.01644(5)	0.02196(8)	0.02822(9)	0.03500(16)	0.0418(2)
U_{12}(Te) (Å^2)	-0.00049(1)	-0.00101(1)	0.00179(1)	-0.00266(2)	0.00355(4)	0.00460(5)	-0.00574(8)	-0.0070(1)
U_{eq}(Te) (Å^2)	0.00312(1)	0.006706(9)	0.01208(2)	0.01786(3)	0.02402(4)	0.03072(5)	0.03824(8)	0.04598(12)
x(Te)	0.183629(6)	0.183232(4)	0.182764(6)	0.182342(9)	0.18188(1)	0.18148(2)	0.18095(3)	0.18057(4)
R_F(obs) (%)	2.47	2.03	1.88	1.89	2.00	2.85	3.07	3.91
wR_F(obs) (%)	5.52	4.16	3.92	4.05	4.61	5.97	5.48	6.76
GoF(obs)	3.07	2.10	2.11	2.26	2.59	3.16	2.93	3.35
R_F(all) (%)	2.54	2.22	2.02	1.95	2.09	3.10	3.23	4.10
wR_F(all) (%)	5.54	4.19	3.96	4.08	4.66	6.03	5.54	6.81
GoF(all)	3.02	2.02	2.04	2.21	2.53	2.92	2.79	3.13

Supplementary Table 6 | Refinement details with the vacancy model of InTe based on the single-crystal synchrotron X-ray diffraction data at 25-700 K. The occupancies of the In^{3+} and Te sites were set to unity.

T (K)	25	100	200	300	400	500	600	700
a (Å)	8.3685(3)	8.3802(3)	8.3996(1)	8.4224(1)	8.4458(2)	8.4697(2)	8.5004(3)	8.5249(3)
c (Å)	7.1212(2)	7.1212(2)	7.1260(2)	7.1339(2)	7.1417(2)	7.1500(2)	7.1562(4)	7.1687(5)
$U_{11}(In^{1+})$ (Å^2)	0.00602(3)	0.01509(4)	0.02780(9)	0.04119(17)	0.0552(3)	0.0700(4)	0.0875(7)	0.1039(10)
$U_{33}(In^{1+})$ (Å^2)	0.02756(11)	0.04353(16)	0.0610(3)	0.0804(5)	0.1007(9)	0.1222(13)	0.138(2)	0.165(3)
$U_{eq}(In^{1+})$ (Å^2)	0.01320(4)	0.02457(6)	0.03885(11)	0.05425(18)	0.0704(3)	0.0874(5)	0.1045(8)	0.1242(10)
$U_{11}(In^{3+})$ (Å^2)	0.00400(2)	0.00860(1)	0.01547(3)	0.02286(4)	0.03075(8)	0.03930(9)	0.04894(17)	0.0592(2)
$U_{33}(In^{3+})$ (Å^2)	0.00213(3)	0.00420(2)	0.00757(3)	0.01124(5)	0.01501(7)	0.01937(8)	0.02414(15)	0.0288(2)
$U_{eq}(In^{3+})$ (Å^2)	0.00338(1)	0.007131(9)	0.01284(2)	0.01898(3)	0.02550(4)	0.03266(5)	0.04068(9)	0.04908(13)
U_{11}(Te) (Å^2)	0.00341(2)	0.00705(1)	0.01263(2)	0.01863(4)	0.02512(6)	0.03206(7)	0.03998(13)	0.04830(18)
U_{33}(Te) (Å^2)	0.00284(2)	0.00615(2)	0.01113(3)	0.01649(4)	0.02202(7)	0.02829(8)	0.03511(15)	0.0419(2)
U_{12}(Te) (Å^2)	0.000489(7)	-0.00101(1)	0.00179(1)	-0.00266(2)	0.00354(3)	0.00460(4)	-0.00573(8)	-0.0070(1)
U_{eq}(Te) (Å^2)	0.00322(1)	0.006748(7)	0.01213(1)	0.01792(2)	0.02409(4)	0.03080(4)	0.03836(8)	0.04616(11)
x(Te)	0.183634(5)	0.183236(4)	0.182769(5)	0.182346(8)	0.18189(1)	0.18148(2)	0.18095(3)	0.18057(4)
Occupancy (In^{1+})	0.901(2)	0.9189(19)	0.918(3)	0.932(3)	0.929(5)	0.921(5)	0.937(7)	0.906(8)
R_F(obs) (%)	2	1.81	1.65	1.73	1.81	2.63	2.87	3.61
wR_F(obs) (%)	4.12	3.39	3.2	3.54	4.1	5.51	5.17	6.07
GoF(obs)	2.29	1.72	1.73	1.97	2.31	2.91	2.76	3.01
R_F(all) (%)	2.06	2	1.8	1.79	1.91	2.88	3.04	3.8
wR_F(all) (%)	4.14	3.43	3.25	3.56	4.16	5.57	5.23	6.11
GoF(all)	2.26	1.65	1.67	1.93	2.26	2.7	2.64	2.81

Supplementary Table 7 | Refinement details with the two interstitial model of InTe based on the single-crystal synchrotron X-ray diffraction data collected at 25-200 K.

T (K)	25	100	200
a (Å)	8.3685(3)	8.3802(3)	8.3996(1)
c (Å)	7.1212(2)	7.1212(2)	7.1260(2)
$U_{11}(In^{1+})$ (Å^2)	0.006051(16)	0.01491(2)	0.02758(6)
$U_{33}(In^{1+})$ (Å^2)	0.02708(6)	0.04253(9)	0.0589(2)
$U_{eq}(In^{1+})$ (Å^2)	0.01306(2)	0.02412(3)	0.03804(7)
$U_{11}(In^{3+})$ (Å^2)	0.004020(11)	0.008609(9)	0.015495(19)
$U_{33}(In^{3+})$ (Å^2)	0.002103(16)	0.004223(12)	0.00761(2)
$U_{eq}(In^{3+})$ (Å^2)	0.003381(7)	0.007147(6)	0.012867(11)
U_{11}(Te) (Å^2)	0.003422(10)	0.007066(7)	0.012660(16)
U_{33}(Te) (Å^2)	0.002866(14)	0.006159(10)	0.01115(2)
U_{12}(Te) (Å^2)	0.000489(5)	0.001005(4)	0.001795(8)
U_{eq}(Te) (Å^2)	0.003237(7)	0.006764(5)	0.012158(10)
$U_{11}(In_i(1,2))$ (Å^2)	0.0049(2)	0.0101(3)	0.0188(6)
$U_{33}(In_i(1,2))$ (Å^2)	0.0041(5)	0.0101(5)	0.0257(14)
$U_{eq}(In_i(1,2))$ (Å^2)	0.00467(19)	0.0101(2)	0.0211(6)
$z(In_i(1))$	0.5463(4)	0.5463	0.5463
$z(In_i(2))$	0.6369(4)	0.6369	0.6369
x(Te)	0.183636(3)	0.183240(2)	0.182777(4)
Occupancy (In^{1+})	0.8948(12)	0.8948	0.8948
Occupancy (In^{3+})	1	1	1
Occupancy (Te)	1	1	1
Occupancy ($In_i(1)$)	0.0156(5)	0.0156	0.0156
Occupancy ($In_i(2)$)	0.0155(5)	0.0155	0.0155
R_F(obs) (%)	1.27	1.24	1.24
wR_F(obs) (%)	2.48	2.19	2.48
GoF(obs)	1.38	1.11	1.34
R_F(all) (%)	1.33	1.43	1.39
wR_F(all) (%)	2.51	2.25	2.54
GoF(all)	1.37	1.08	1.31

Supplementary Table 8 | Details of the MEM calculations on InTe performed in BayMEM[7] at 25-700 K. The MEM calculations were conducted with a uniform (flat) prior density as well as structure factors from the full occupancy model. For each temperature, the residual density analysis was conducted and the details of the MEM calculation with the optimal χ^2 are shown in this table.

T (K)	25	100	200	300	400	500	600	700
χ^2	0.02	0.01	0.02	0.04	0.01	0.05	0.10	0.10
R^2	0.95	0.94	0.87	0.94	0.90	0.91	0.91	0.92
Entropy	-2827.8	-2572.6	-2344.9	-2173.6	-2084.7	-1844.3	-1713.1	-1596.6
R/R_w (%)	0.24/0.26	0.20/0.21	0.26/0.28	0.37/0.37	0.19/0.18	0.57/0.46	0.69/0.63	0.80/0.69
$\Delta\rho_{max}$ (e Å^{-3})	0.79	0.76	0.69	0.81	0.33	0.53	0.57	0.50
$\Delta\rho_{min}$ (e Å^{-3})	-0.57	-0.49	-0.40	-0.38	-0.18	-0.66	-0.63	-0.71
Cycles No.	227679	150466	90073	69491	146673	75185	20704	18926

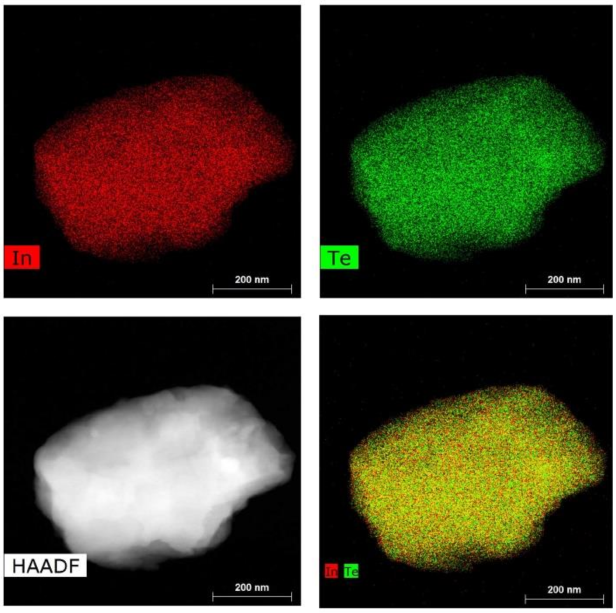

Supplementary Fig. 1 | STEM-EDS elemental mapping of a small crystal extracted from the as-grown large InTe single crystal.

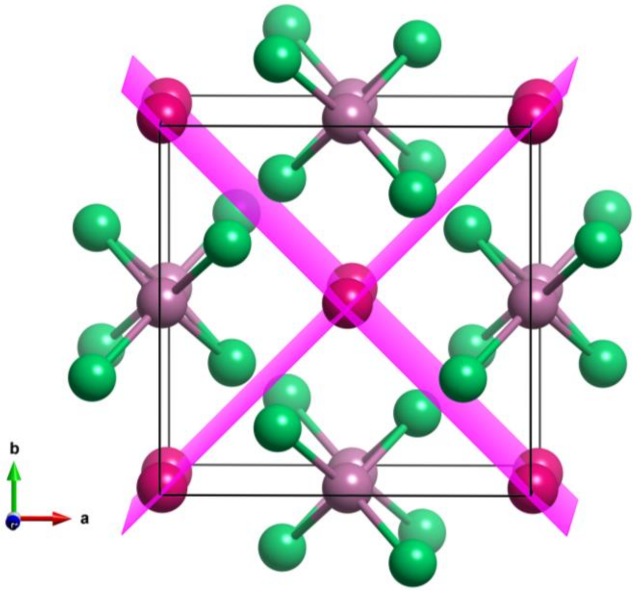

Supplementary Fig. 2 | Illustration of the two perpendicular cleavage planes (*hh*0) for determining the *c* direction in InTe. Due to the four-fold rotational symmetry of the *I*4/*mcm* space group, the two perpendicular cleavage planes of (*hh*0) and ($h\bar{h}0$) are symmetry-equivalent. Therefore, the intersection line of the two perpendicular cleavage planes of (*hh*0) defines the *c* axis.

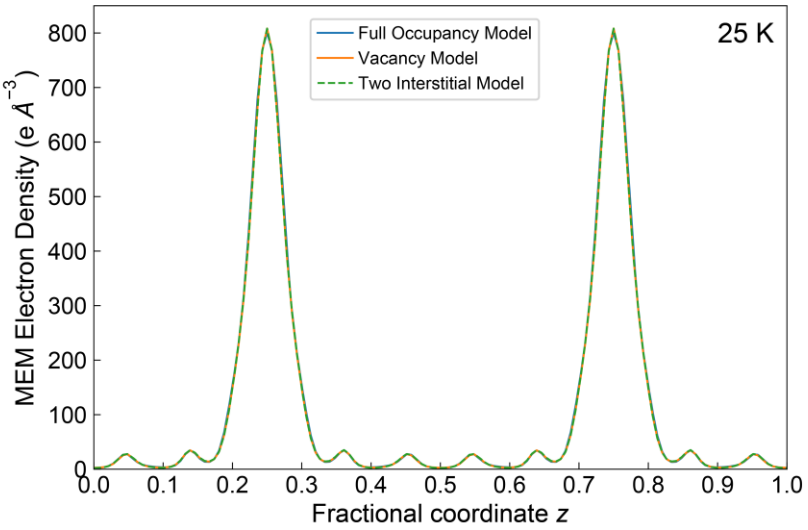

Supplementary Fig. 3 | Comparison of 1D MEM electron density profiles with different structure models. The test of MEM calculations at 25 K is based on the structure factors extracted with the flat prior density as well as $\chi^2 = 0.02$.

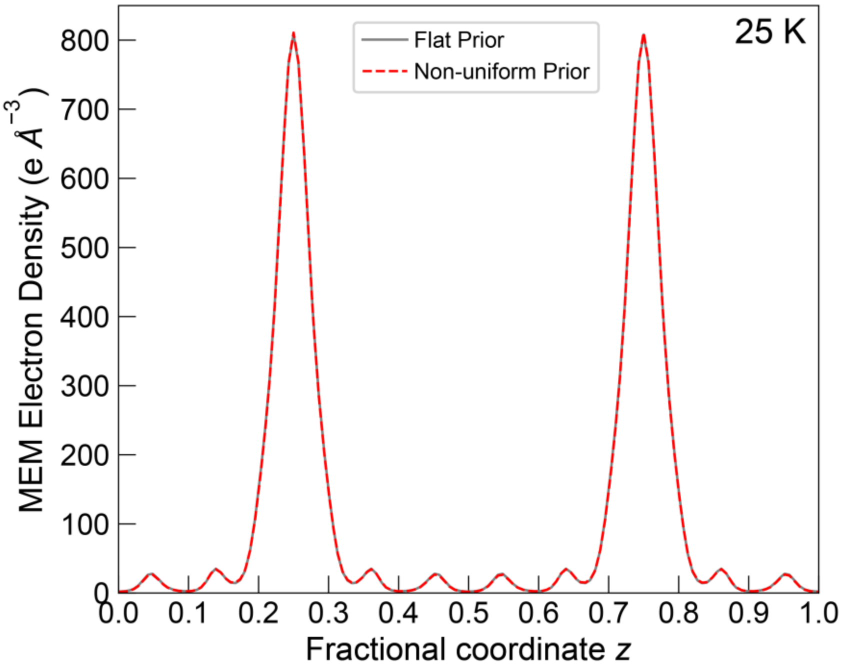

Supplementary Fig. 4 | Comparison of 1D MEM electron density profiles with flat prior and non-uniform prior density. The test of MEM calculations at 25 K is based on the structure factors extracted with the full occupancy model as well as $\chi^2 = 0.02$.

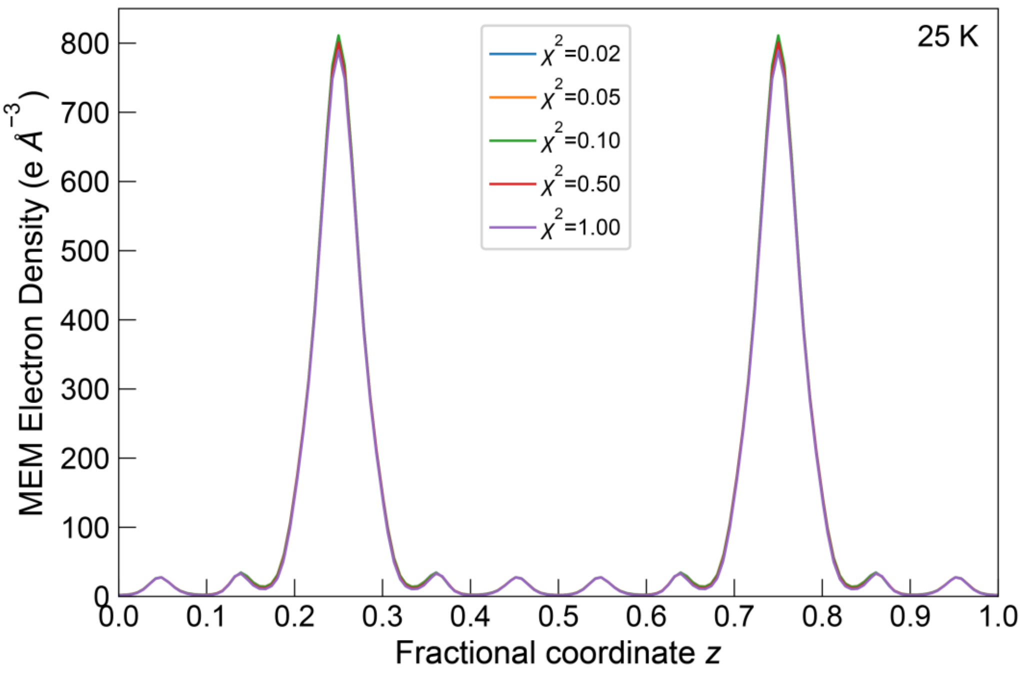

Supplementary Fig. 5 | Comparison of 1D MEM electron density profiles with different stopping criteria χ^2. The test of MEM calculations at 25 K is based on the flat prior density and structure factors extracted with the full occupancy model.

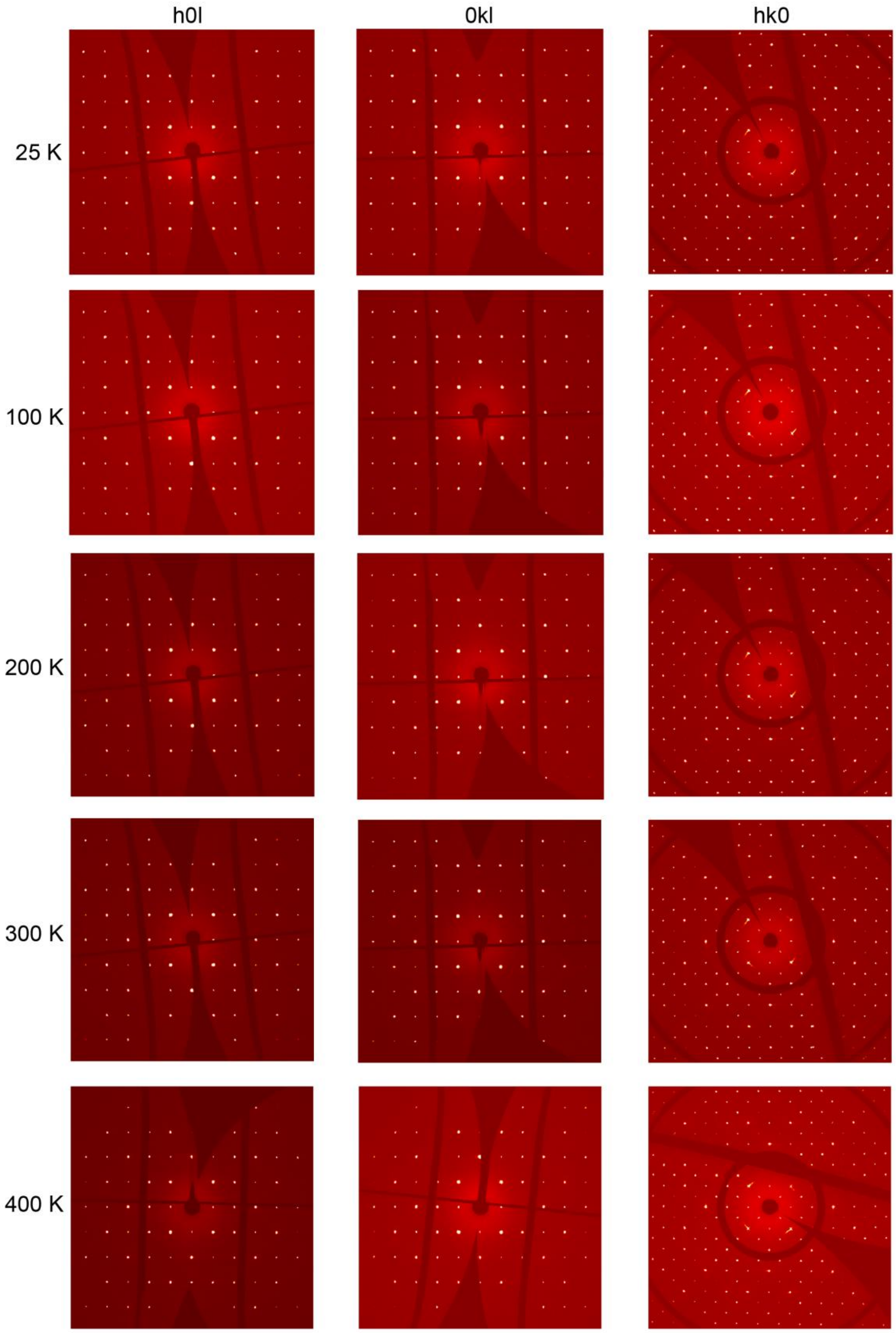

Supplementary Fig. 6 | Precession images of single-crystal synchrotron X-ray diffraction data at 25-400 K. Precession images of *h*0*l*, 0*kl*, and *hk*0 planes of InTe single crystal at 25-400 K with the single-crystal synchrotron X-ray diffraction data collected at the BL02B1 beamline from SPring-8 using a photon energy of 50.00 keV.

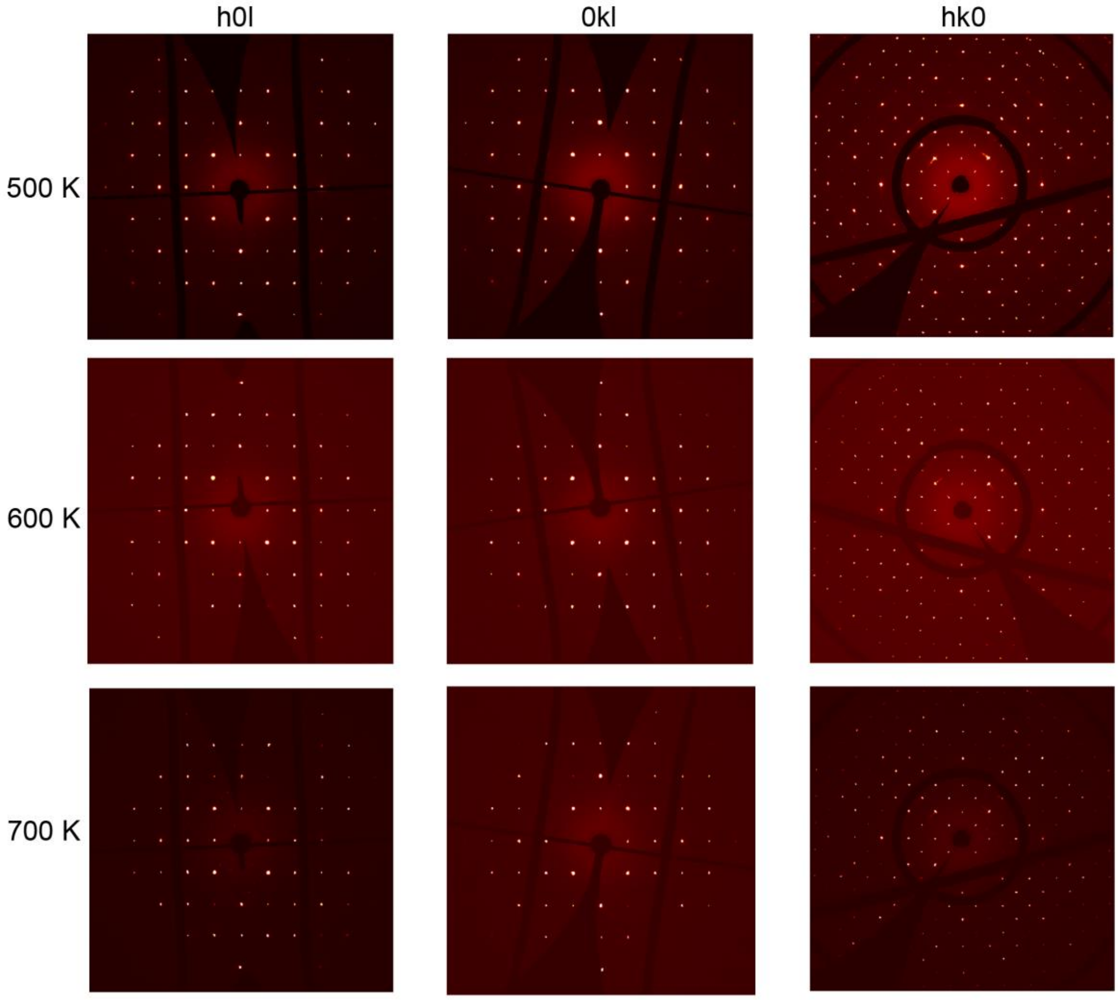

Supplementary Fig. 7 | Precession images of single-crystal synchrotron X-ray diffraction data at 500-700 K. Precession images of *h*0*l*, 0*kl*, and *hk*0 planes of InTe single crystal at 500-700 K with the single-crystal synchrotron X-ray diffraction data collected at the BL02B1 beamline from SPring-8 using a photon energy of 50.00 keV.

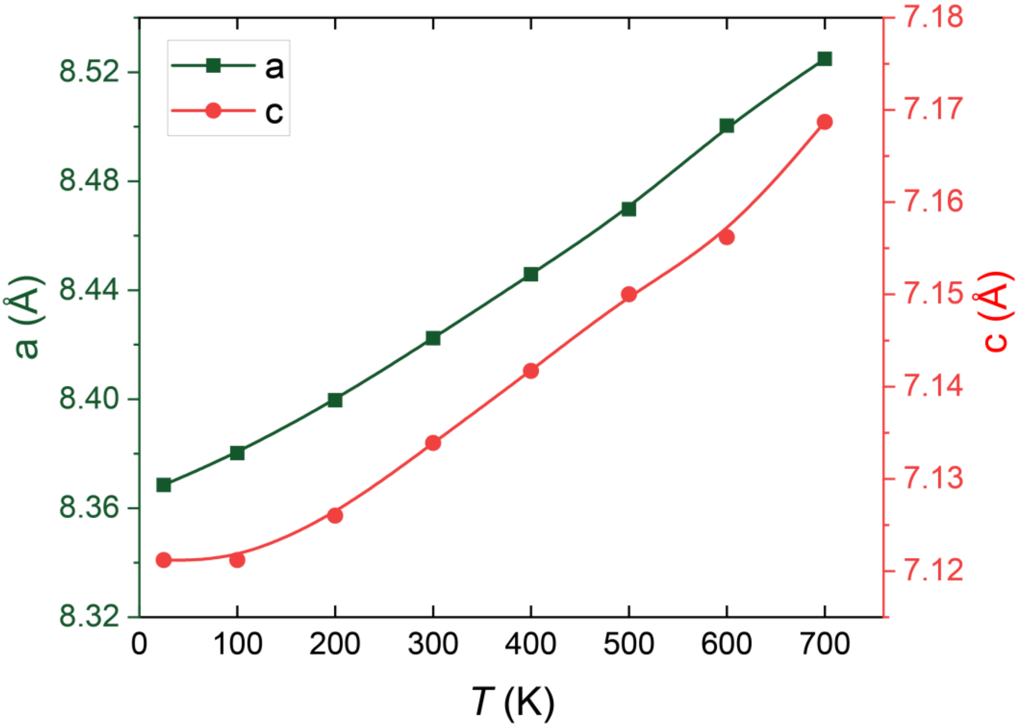

Supplementary Fig. 8 | Temperature dependence of lattice parameters of InTe extracted from multi-tempearture single-crystal synchrotron X-ray diffraction data. The uncertainty is smaller than the symbol size. Due to the wrong calibration of the wavelengh for the 500-700K data collection, the lattice parameters at 500-700 K were manually shifted to fit with those at 25-400 K.

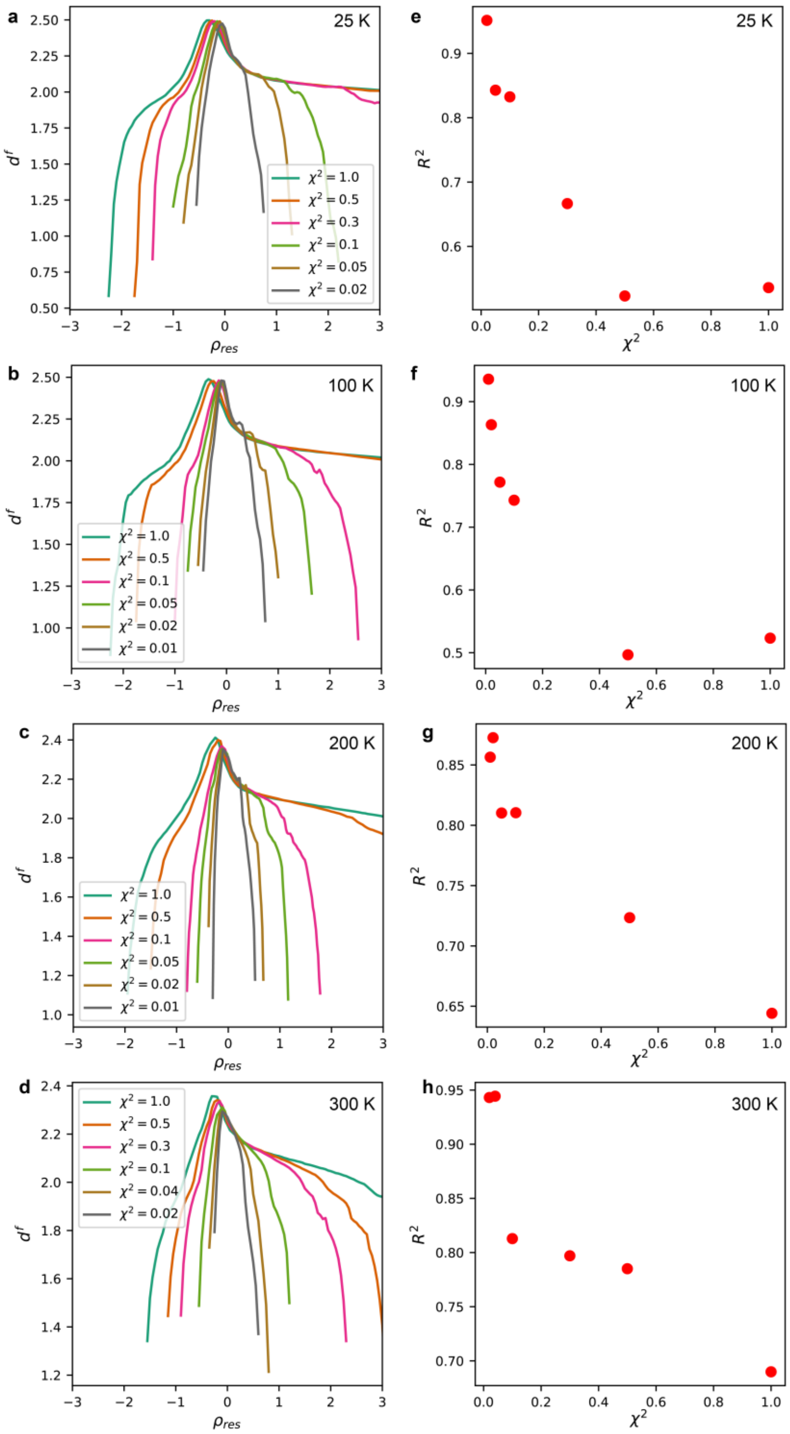

Supplementary Fig. 9 | Residual density analysis for MEM calculations of InTe at 25-300K. (**a-d**) The fractal dimension distributions as a function of the residual density ρ_{res} for a series of different stopping criteria χ^2 at 25-300 K. (**e-h**) Coefficients of determination R^2, obtained by fitting the fractal dimension distributions to a parabolic function, as a functional of χ^2 at 25-300 K.

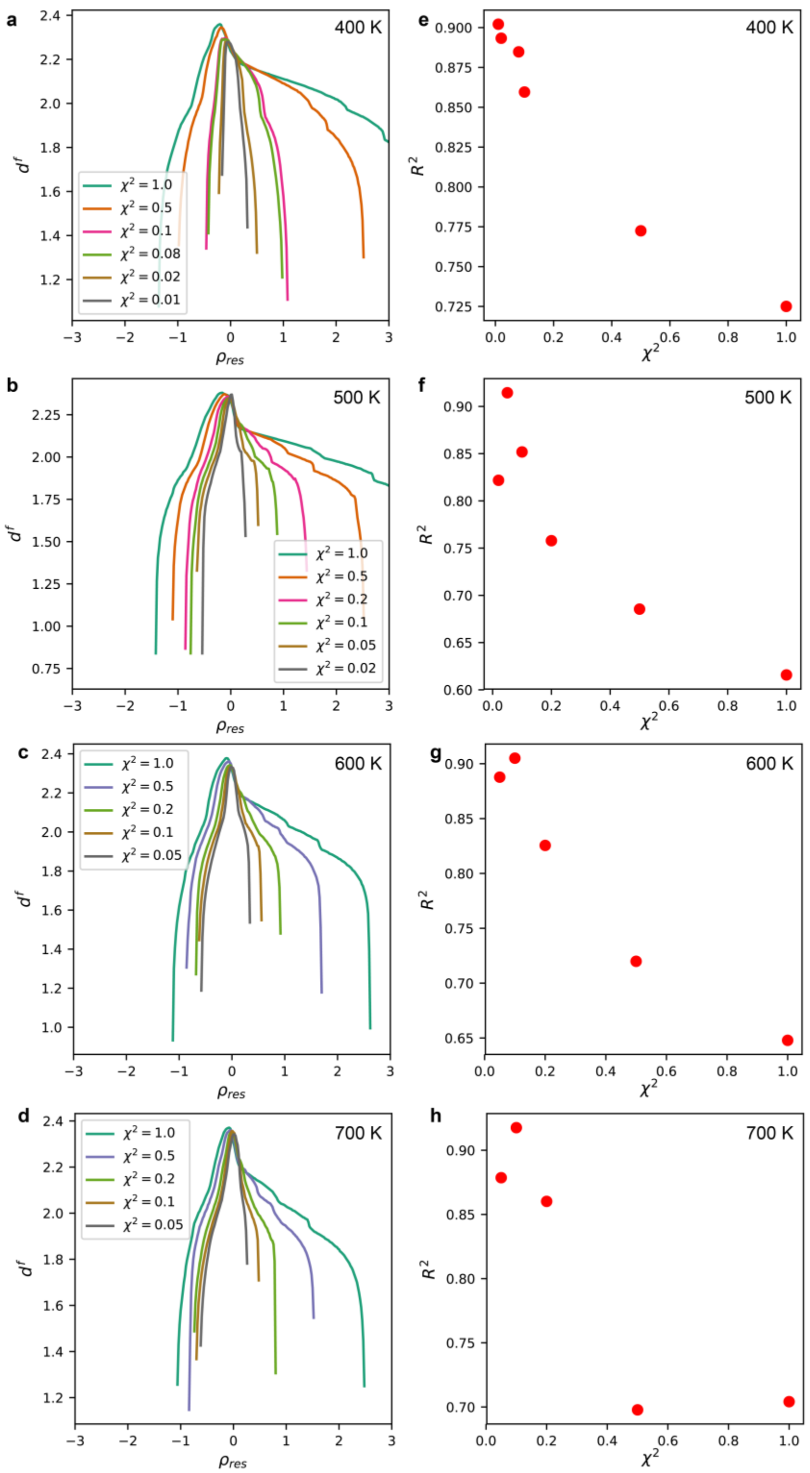

Supplementary Fig. 10 | Residual density analysis for MEM calculations of InTe at 400-700K. (**a-d**) The fractal dimension distributions as a function of the residual density ρ_{res} for a series of different stopping criteria χ^2 at 400-700 K. (**e-h**) Coefficients of determination R^2, obtained by fitting the fractal dimension distributions to a parabolic function, as a functional of χ^2 at 400-700 K.

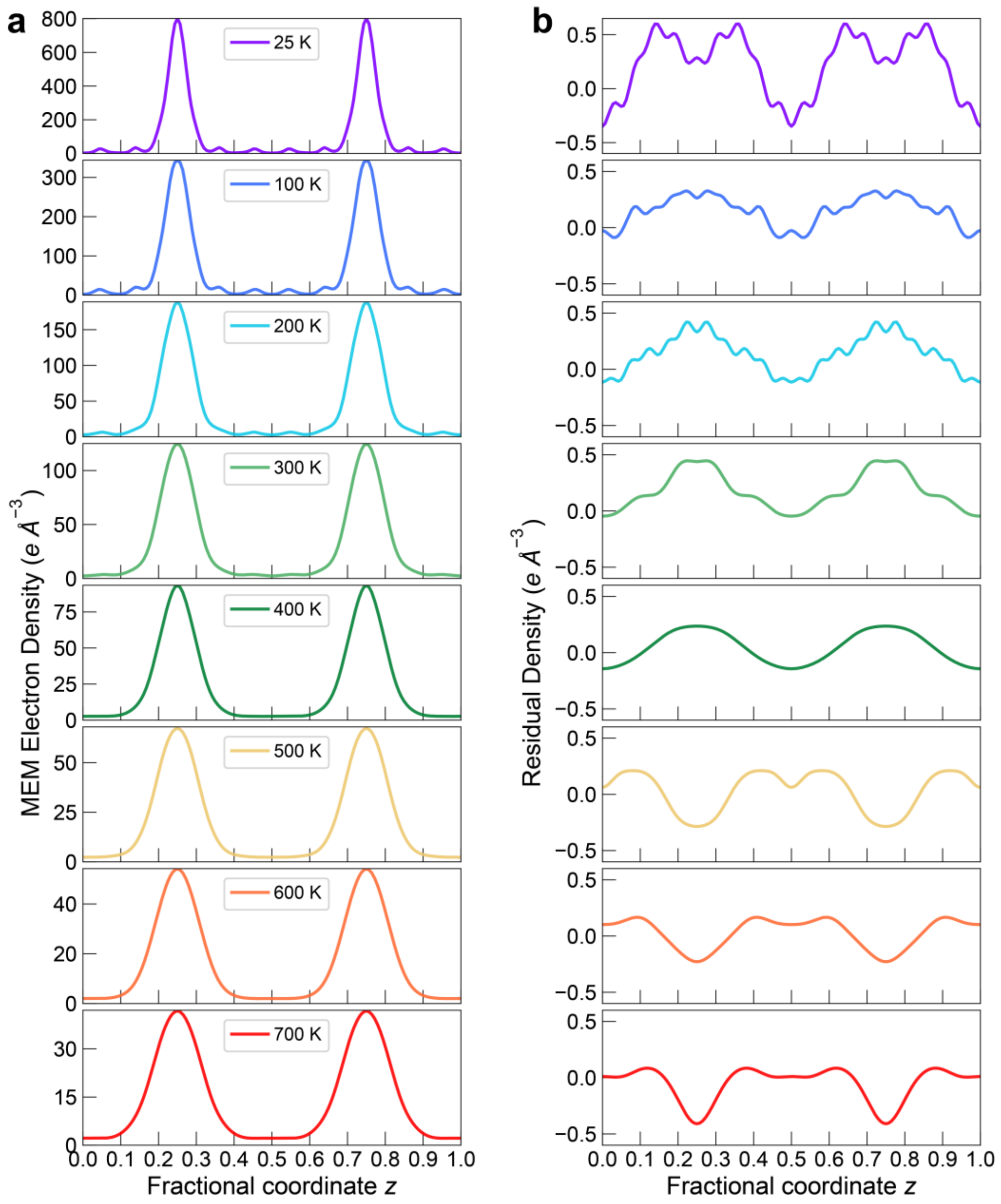

Supplementary Fig. 11 | One-dimensional MEM electron density and residual density profiles at 25-700 K through the In^{1+} atoms along the [001] direction. The origin of the line plot is placed at (*x* = 0.5, *y* = 0.5, *z* = 0).

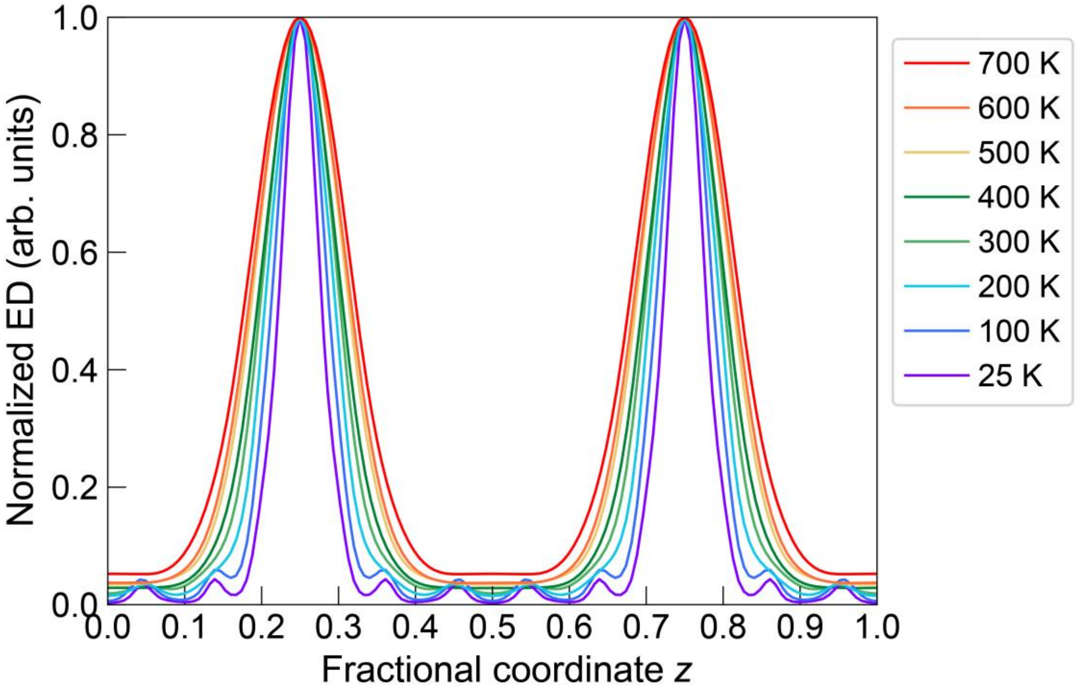

Supplementary Fig. 12 | Normalized one-dimensional MEM electron density profiles at 25-700 K through the In^{1+} atoms along the [001] direction. The electron density profiles are normalized by the peak values at the In^{1+} sites for better comparison. The origin of the line plot is placed at (x = 0.5, y = 0.5, z = 0).

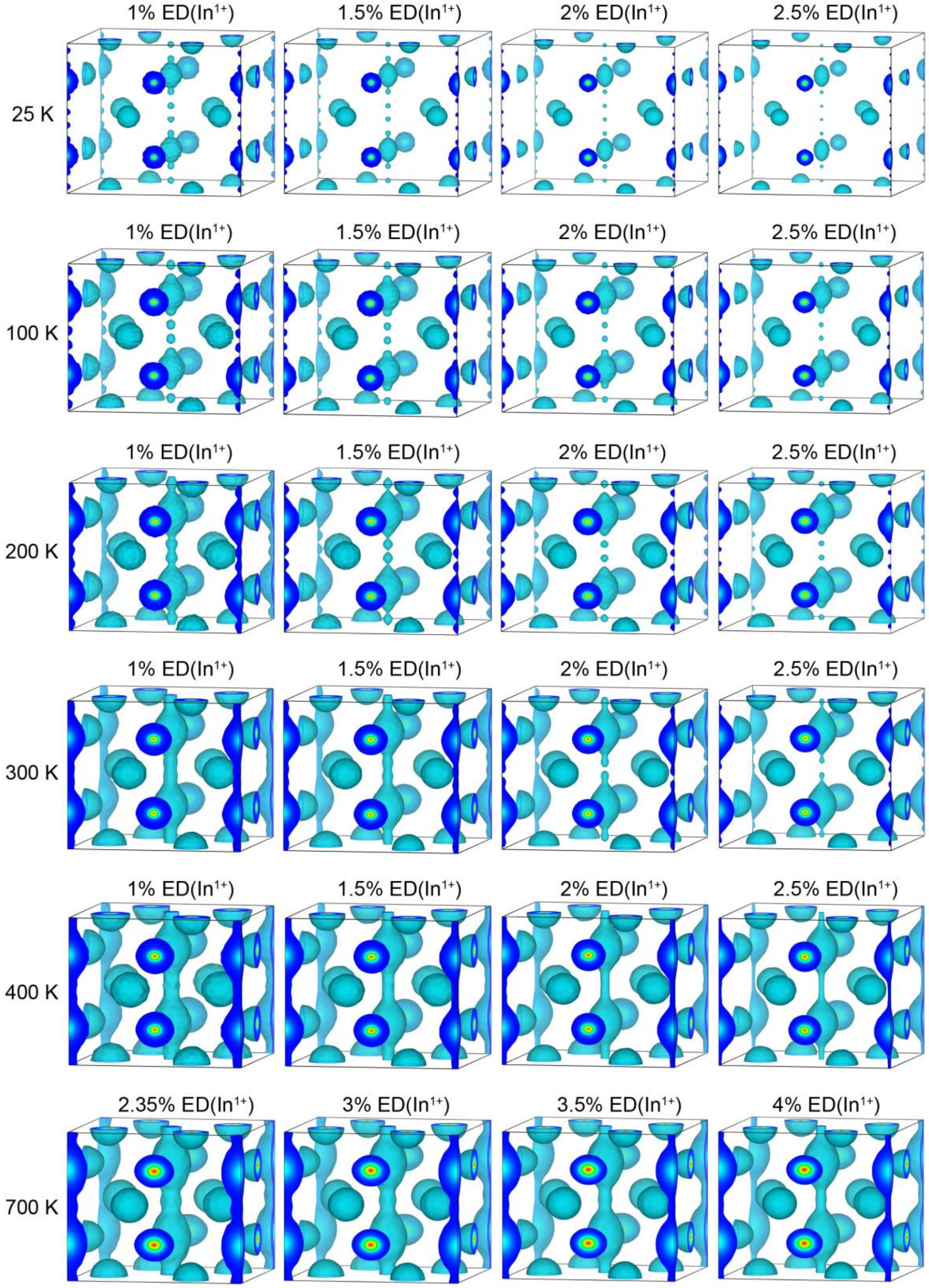

Supplementary Fig. 13 | 3D MEM electron density surfaces of InTe at 25-400 and 700 K with a series of isovalues. 2% ED(In^{1+}) denotes the isosurface value being 2% of the electron density peak value at the In^{1+} site.

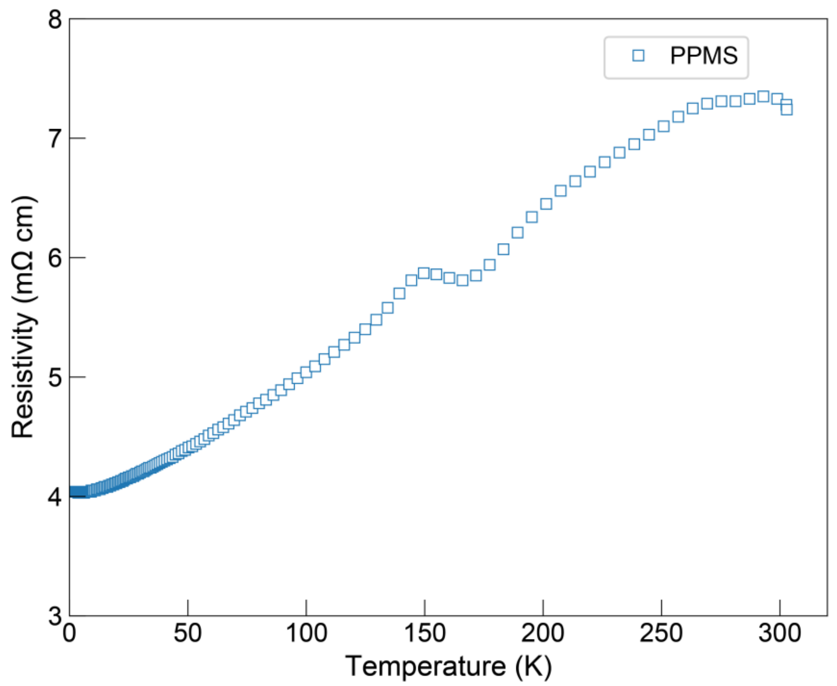

Supplementary Fig. 14 | Temperature-dependent resistivity of InTe single crystal along the *c* axis. The uncertainty is smaller than the symbol size.

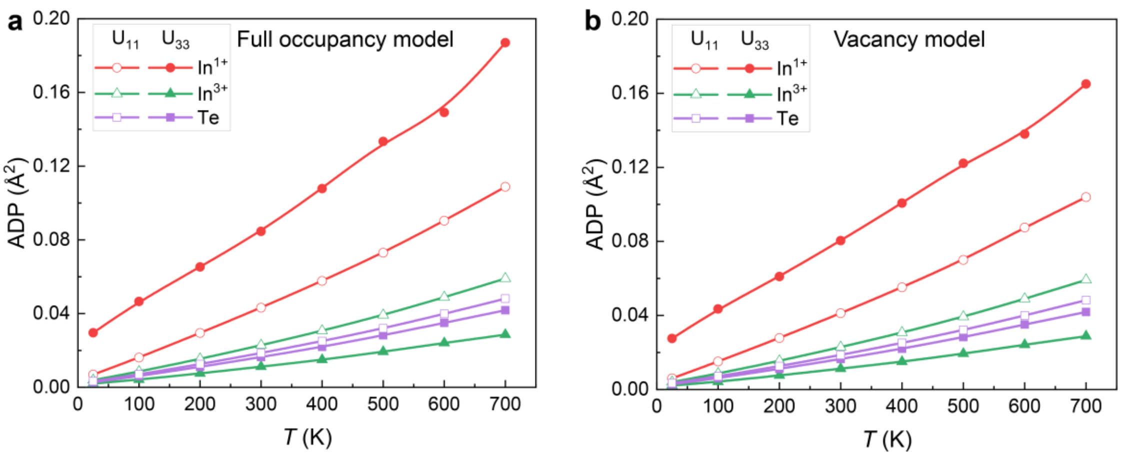

Supplementary Fig. 15 | Temperature-dependent anisotropic atomic displacement parameters (ADPs) in InTe with the full occupancy model and vacancy model. Anisotropic ADPs of three nonequivalent atoms (i.e., In^{1+}, In^{3+}, and Te) in InTe with (**a**) the full occupancy model and (**b**) vacancy model. The U_{33} values of the In^{1+} atom at high temperatures do not extrapolate to zero at 0 K, a clear indication of the atomic disorder of the In^{1+} along the *c* direction.

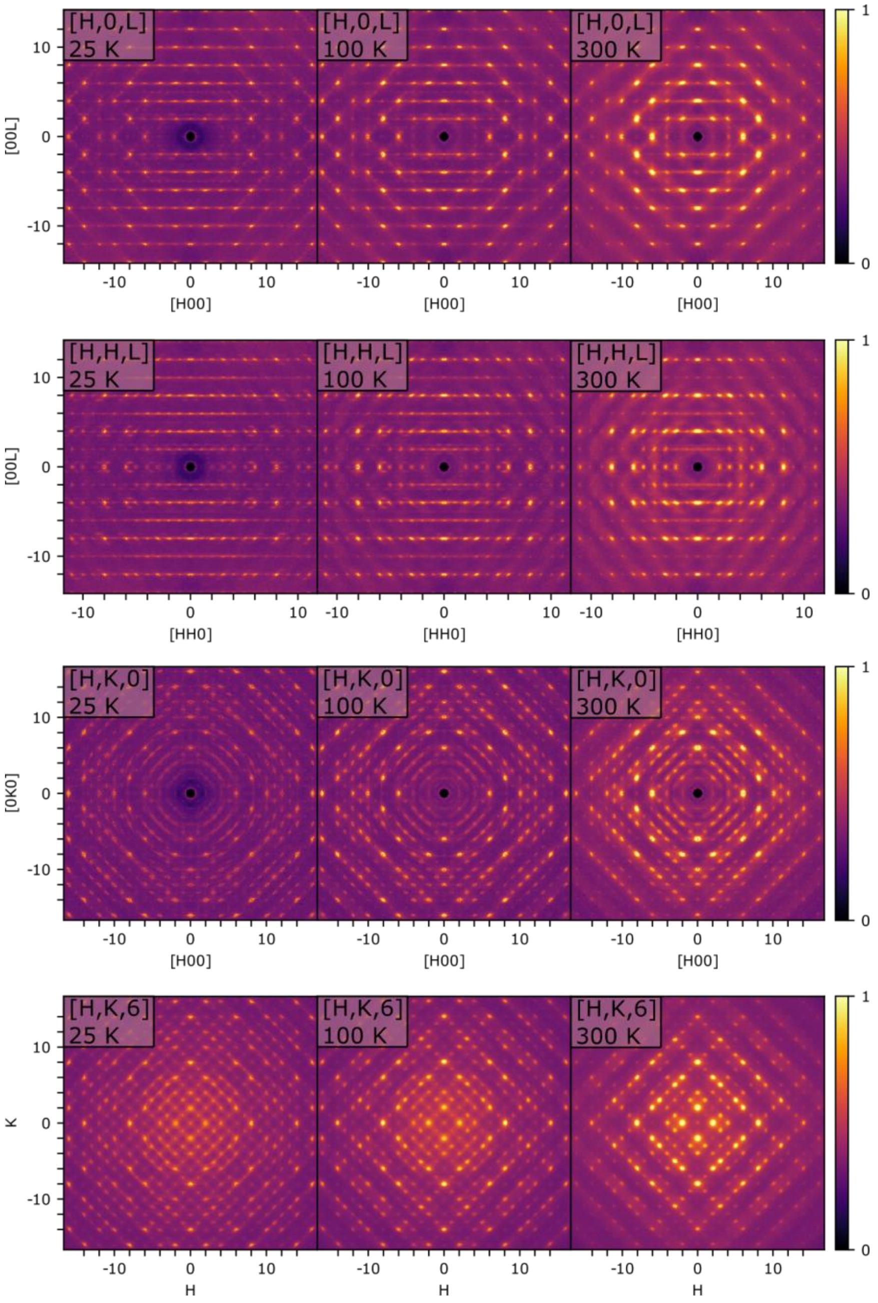

Supplementary Fig. 16 | Single-crystal diffuse X-ray scattering of InTe. Single-crystal diffuse X-ray scattering of InTe in the [*H*,0,*L*], [*H*,*H*,*L*], [*H*,*K*,0] and [*H*,*K*,6] planes at 25, 100 and 300 K. The diffuse scattering shown is before filling the masked regions at the Bragg peaks. The diffuse scattering at 25 K mainly consists of 2D layers for even values of *L* (except *L*=0), but there are also diagonal lines between some of the layers, best seen in the *H*0*L* plane. During heating the layers of scattering become weaker, and more broad 3D diffuse scattering features become stronger. Color bars represent the intensity in arbitrary units.

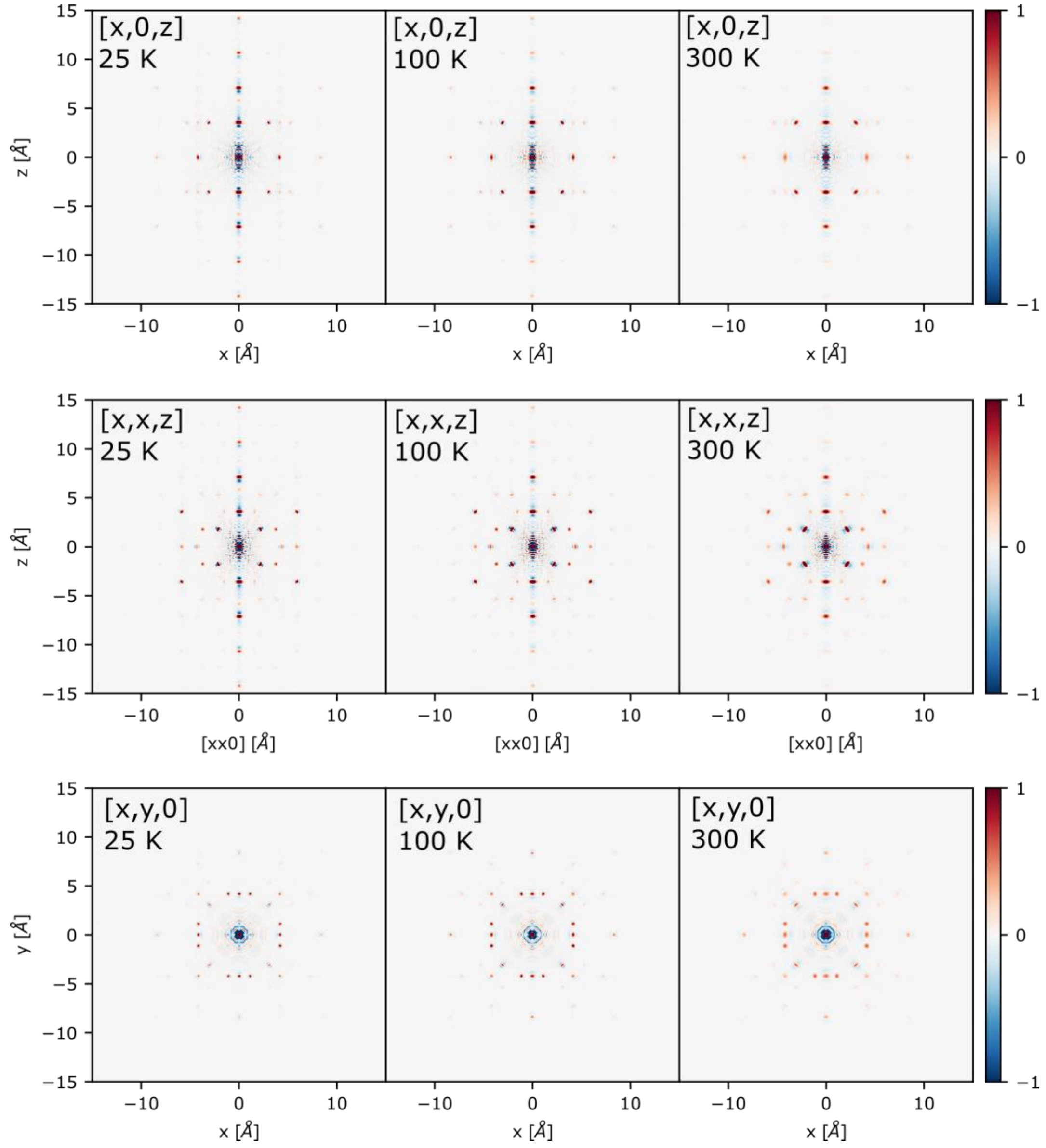

Supplementary Fig. 17 | 3D-ΔPDF of InTe. 3D-ΔPDF of InTe in the [*x*,0,*z*], [*x*,*x*,*z*] and [*x*,*y*,0] planes at 25, 100 and 300 K. Color bars represent the 3D-ΔPDF in arbitrary units.

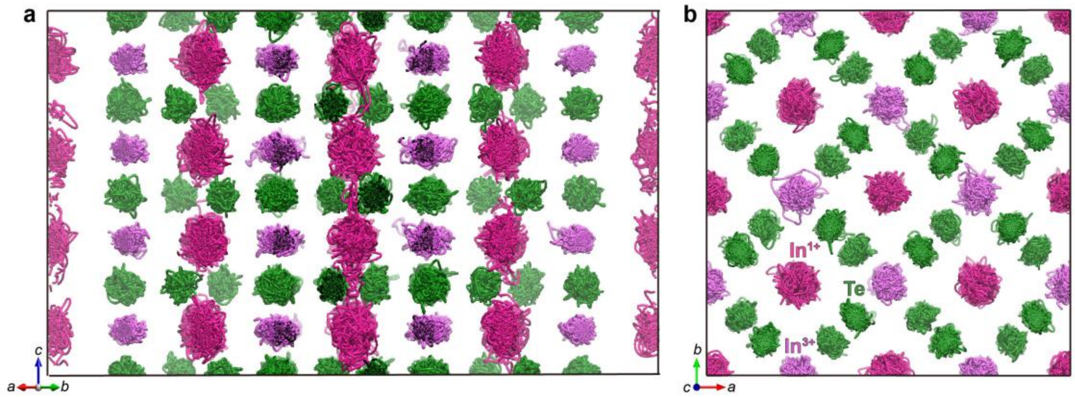

Supplementary Fig. 18 | *Ab initio* molecular dynamics trajectories of all atoms in $In_{0.984}Te$ ($In_{63}Te_{64}$) at 700 K. Molecular dynamics simulation trajectories of indium and tellurium atoms in $In_{0.984}Te$ projected along the [110] (**a**) and [001] (**b**) directions.

Supplementary References

1. Schubert, K., Anderko, K., Kluge, M., Beeskow, H., Ilschner, M., Doerre, E. & Esslinger, P. Strukturuntersuchung der Legierungsphasen Cu_2Te, CuTe, Cu_3Sb, InTe, Bi_2Se_3, Pd_5Sb_3 und Pd_5Bi_3. *Naturwissenschaften* **40,** 269-269 (1953).
2. Hogg, J. H. C. & Sutherland, H. H. Indium telluride. *Acta Crystallogr. B* **32,** 2689-2690 (1976).
3. Willis, B. T. M. & Pryor, A. W. *Thermal Vibrations in Crystallography* (Cambridge University Press, Cambridge, 1975).
4. Sales, B. C., Chakoumakos, B. C., Mandrus, D. & Sharp, J. W. Atomic displacement parameters and the lattice thermal conductivity of clathrate-like thermoelectric compounds. *J. Solid State Chem.* **146,** 528-532 (1999).
5. Bindzus, N. & Iversen, B. B. Maximum-entropy-method charge densities based on structure-factor extraction with the commonly used Rietveld refinement programs GSAS, FullProf and Jana2006. *Acta Crystallogr. A* **68,** 750-762 (2012).
6. Meindl, K. & Henn, J. Foundations of residual-density analysis. *Acta Crystallogr. A* **64,** 404-418 (2008).
7. van Smaalen, S., Palatinus, L. & Schneider, M. The maximum-entropy method in superspace. *Acta Cryst. A* **59,** 459-469 (2003).
